# Supplementary material for: Invariant and smooth limit of discrete geometry folded from bistable origami leading to multistable metasurfaces
Source: Nat Commun. 2019 Sep 17;10:4238. doi: 10.1038/s41467-019-11935-x (PMC6748981; doi:10.1038/s41467-019-11935-x)
Supplement: Supplementary file 1 — Supplementary Information [file 41467_2019_11935_MOESM1_ESM.pdf]

SUPPLEMENTARY INFORMATION

Invariant and smooth limit of discrete geometry folded from bistable origami leading to multistable tessellations

Liu et al.

Supplementary Note 1: Geometry of corrugations

There are two options to triangulate the hypar pattern [1]: one is shown in Fig.1(a), known as the alternating asymmetric triangulation, and the other is shown in Fig.1(b), known as the asymmetric triangulation. Although we find that both triangulation schemes yield the same results, we report the derivation based on the alternating asymmetric triangulation because it is kinematically preferred for large folding [1], and it relates closely to the natural bending of hypar panels as we show in our research. In this section we address the geometry of corrugations. We first parametrize a single corrugation by one folding angle $\rho \in [0, \pi]$ and two bending angles $\theta_1, \theta_2 \in [0, \pi]$, considering panel width d and middle ridge length L. For the coupled panels ABCDEF shown in Fig. 1(c), its middle ridge is a folding crease labeled as BE, whose length equals L. For convenience of derivation, we set our coordinate system so that the panel BCFE is on the xy plane and point E coincides the origin. Without loss of generality, we scale the dimension of the structure by L, and then the panel width becomes the relevant dimensionless quantity $w := d/L$. For folded corrugation before twisting, the coordinates of points after scaling are given as:

$$
\begin{gathered}
\mathrm{A}_x = 1 - w, \quad \mathrm{A}_y = w\cos\rho, \quad \mathrm{A}_z = w\sin\rho \\
\mathrm{B}_x = 1, \quad \mathrm{B}_y = 0, \quad \mathrm{B}_z = 0 \\
\mathrm{C}_x = 1 + w, \quad \mathrm{C}_y = -w, \quad \mathrm{C}_z = 0 \\
\mathrm{D}_x = w, \quad \mathrm{D}_y = w\cos\rho, \quad \mathrm{D}_z = w\sin\rho \\
\mathrm{E}_x = 0, \quad \mathrm{E}_y = 0, \quad \mathrm{E}_z = 0 \\
\mathrm{F}_x = -w, \quad \mathrm{F}_y = -w, \quad \mathrm{F}_z = 0
\end{gathered} \tag{1}
$$

For isometric folding of the triangulated model (using alternating asymmetric triangulation), *twisting of a corrugation* is achieved by bending about diagonals of the two panels. For example, the bending of panel ABED is realized by rotating triangle △ADE about the diagonal EA with angle θ_1, as illustrated in Fig. 1(d). Using Rodrigues' rotation formula [2], we obtain the vector $\mathbf{e}_{\mathrm{ED}}$ after rotation by

$$
\mathbf{e}_{\mathrm{ED'}} = \cos\theta_1 \mathbf{e}_{\mathrm{ED}} + \sin\theta_1 (\frac{\mathbf{e}_{\mathrm{EA}}}{\|\mathbf{e}_{\mathrm{EA}}\|} \times \mathbf{e}_{\mathrm{ED}}) + (1 - \cos\theta_1)(\frac{\mathbf{e}_{\mathrm{EA}}}{\|\mathbf{e}_{\mathrm{EA}}\|} \cdot \mathbf{e}_{\mathrm{ED}}) \frac{\mathbf{e}_{\mathrm{EA}}}{\|\mathbf{e}_{\mathrm{EA}}\|}. \tag{2}
$$

Because E' remains the same as E (located at the origin), then we can write the new

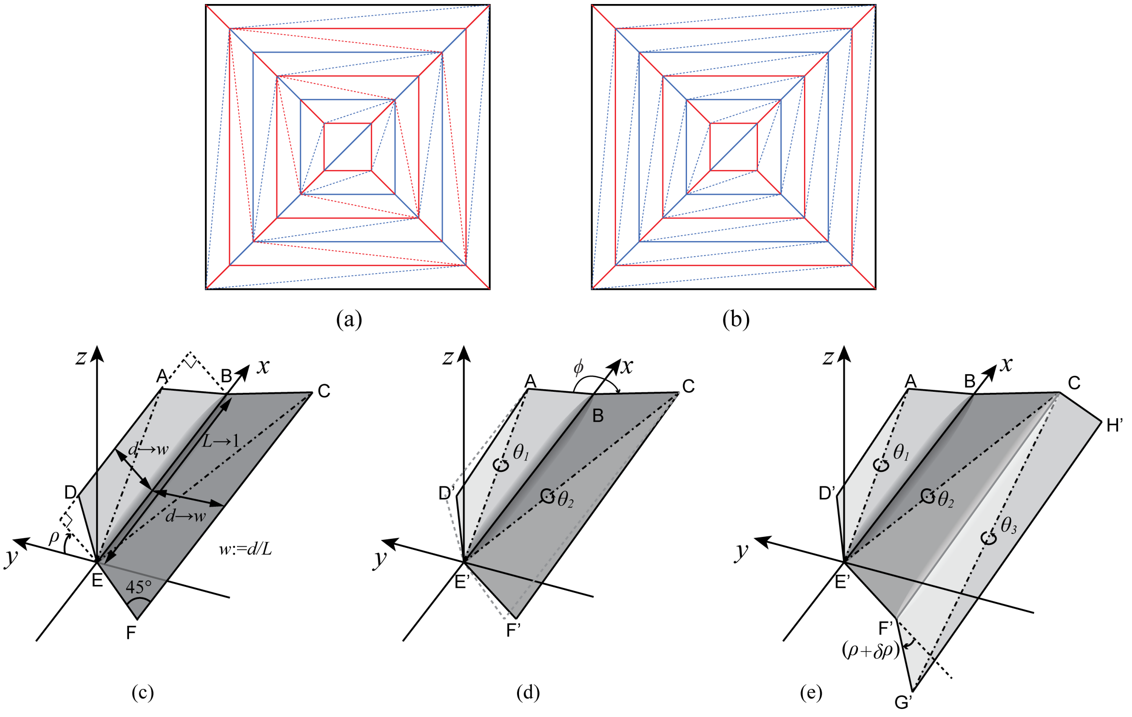

Supplementary Figure 1. Geometry of corrugations for the hypar pattern – dashed lines indicate extra pleats introduced by triangulation. (a) Alternating asymmetric triangulation. (b) Asymmetric triangulation. (c) A folded corrugation before twisting. (d) A folded corrugation after twisting. (e) Twisted configuration of an augmented system considering two adjacent corrugations, where panel BCF'E' is shared.

coordinates of point D as:

$$\begin{aligned}
\text{D'}_x &= \frac{w((2w-1)w\cos\theta_1 - w + 1)}{2(w-1)w+1} \\
\text{D'}_y &= \frac{w\left((2w-1)\sqrt{2(w-1)w+1}\sin\theta_1\sin\rho + \cos\rho((w-1)(2w-1)\cos\theta_1 + w)\right)}{2(w-1)w+1} \\
\text{D'}_z &= \frac{w\left((1-2w)\sqrt{2(w-1)w+1}\sin\theta_1\cos\rho + \sin\rho((w-1)(2w-1)\cos\theta_1 + w)\right)}{2(w-1)w+1}
\end{aligned} \tag{3}$$

Similarly, we derive the new coordinates of point F as:

$$\begin{aligned}
\text{F'}_x &= -\frac{w((2w+1)w\cos\theta_2 + w + 1)}{2w^2+2w+1} \\
\text{F'}_y &= -\frac{w\left((2w^2+3w+1)\cos\theta_2 - w\right)}{2w^2+2w+1} \\
\text{F'}_z &= -\frac{w(2w+1)\sin\theta_2}{\sqrt{2w^2+2w+1}}
\end{aligned} \tag{4}$$

The other points remain unchanged – see Fig. 1(d).

To account for the two reflection symmetries of the entire folded shell, and considering the orthogonality of the two symmetry planes, the normals of the faces △ABC and △D'E'F' must be orthogonal, as illustrated by Fig. 1(d). Accordingly, the normals of the faces △ABC and △D'E'F' are given by:

$$\vec{n}_{\mathrm{ABC}} = (\mathbf{e}_{\mathrm{BA}} \times \mathbf{e}_{\mathrm{BC}})/(2w^2) \tag{5}$$

$$\vec{n}_{\mathrm{D'E'F'}} = (\mathbf{e}_{\mathrm{E'D'}} \times \mathbf{e}_{\mathrm{E'F'}})/(2w^2) \tag{6}$$

respectively. In the limit $w \to 0$, we obtain

$$\lim_{w\to 0} \vec{n}_{\mathrm{D'E'F'}} = \left[\sin(\theta_1 - \theta_2 + \rho),\ \sin\theta_2 - \sin(\theta_1+\rho),\ \cos(\theta_1+\rho) - \cos\theta_2\right]/2, \tag{7}$$

$$\lim_{w\to 0} \vec{n}_{\mathrm{ABC}} = \vec{n}_{\mathrm{ABC}} = \left[\sin\rho,\ \sin\rho,\ 1-\cos\rho\right]/2. \tag{8}$$

Thus the orthogonality condition yields:

$$\lim_{w\to 0} \left(\vec{n}_{\mathrm{ABC}} \cdot \vec{n}_{\mathrm{D'E'F'}}\right) = 0 \implies \tag{9}$$
$$(\cos\rho - 1)(\cos\theta_2 - \cos(\theta_1+\rho)) + \sin\rho(\sin\theta_2 - \sin(\theta_1+\rho)) + \sin\rho\sin(\theta_1-\theta_2+\rho) = 0.$$

The above constraint enforces compatibility around the circumferential direction.

Now let's consider compatibility in the radial direction. We augment the structure by considering the next corrugation that shares the panel BCFE with the one we just analyzed – see Fig. 1(e). Notice the interesting fact that the mountain-valley assignment is opposite for crease lines BE and CF. After the twist, to ensure that the two corrugations comply with the global symmetries, the normals of △E'F'G' and △BCH' must be parallel with the normals of face △D'E'F' and △ABC, respectively. Realizing that $\mathbf{e}_{\mathrm{E'B}}$ ($= \mathbf{e}_{\mathrm{EB}}$) is parallel to $\mathbf{e}_{\mathrm{F'C}}$ in the limit, the following constraints must be satisfied:

$$\lim_{w\to 0} \left(\vec{n}_{\mathrm{D'E'F'}} \cdot \mathbf{e}_{\mathrm{EB}}\right) = \lim_{w\to 0} \left(\vec{n}_{\mathrm{E'F'G'}} \cdot \mathbf{e}_{\mathrm{F'C}}\right), \tag{10}$$

$$\lim_{w\to 0} \left(\vec{n}_{\mathrm{ABC}} \cdot \mathbf{e}_{\mathrm{EB}}\right) = \lim_{w\to 0} \left(\vec{n}_{\mathrm{BCH'}} \cdot \mathbf{e}_{\mathrm{F'C}}\right), \tag{11}$$

where

$$\vec{n}_{\mathrm{BCH'}} = (\mathbf{e}_{\mathrm{CH'}} \times \mathbf{e}_{\mathrm{CB}})/(2w^2),$$
$$\vec{n}_{\mathrm{E'F'G'}} = (\mathbf{e}_{\mathrm{F'G'}} \times \mathbf{e}_{\mathrm{F'E'}})/(2w^2). \tag{12}$$

Again, in the limit of $w \to 0$, the two corrugations essentially yield the same angular relationships, and the small variation in folding angle $\delta\rho$ vanishes along with w – see Fig. 1(e). Therefore, one infers that the twisted corrugation BCH'E'F'G' is simply ABCD'E'F' flipped. As a result, we obtain the following identities:

$$\lim_{w\to 0}\left(\vec{n}_{\mathrm{BCH'}}\cdot\mathbf{e}_{\mathrm{F'C}}\right)=\lim_{w\to 0}\left(\vec{n}_{\mathrm{D'E'F'}}\cdot\mathbf{e}_{\mathrm{EB}}\right), \tag{13}$$

$$\lim_{w\to 0}\left(\vec{n}_{\mathrm{E'F'G'}}\cdot\mathbf{e}_{\mathrm{F'C}}\right)=\lim_{w\to 0}\left(\vec{n}_{\mathrm{ABC}}\cdot\mathbf{e}_{\mathrm{EB}}\right). \tag{14}$$

Thus,

$$\lim_{w\to 0}\left(\vec{n}_{\mathrm{ABC}}\cdot\mathbf{e}_{\mathrm{EB}}\right)=\lim_{w\to 0}\left(\vec{n}_{\mathrm{D'E'F'}}\cdot\mathbf{e}_{\mathrm{EB}}\right), \tag{15}$$

which leads to,

$$\sin\rho=\sin(\theta_1-\theta_2+\rho). \tag{16}$$

Considering the definition ranges of the angles and observations of physical models, we should reduce the above identity to the simple fact that in the limit ($w \to 0$):

$$\theta_1=\theta_2, \tag{17}$$

which leads to $\angle$ABC = $\angle$D'E'F', and hence $\triangle$ABC $\cong$ $\triangle$D'E'F'. Substituting Eq. (17) to Eq. (9), we obtain

$$(1-2\cos\theta_1+\cos\rho)\sin^2(\rho/2)=0. \tag{18}$$

For $\rho > 0$, the above expression reduces to:

$$\cos\theta_1=\cos^2(\rho/2). \tag{19}$$

Next, we derive the amount of twisting of a corrugation in terms of ρ, θ_1 and θ_2. The axis of twisting is labeled as $\mathbf{e}_{\mathrm{MN}}$ in Fig. 2 of the main text, where M can be regarded as the middle point of AC, and N can be regarded as the middle point of D'F'. Although $\mathbf{e}_{\mathrm{MN}}$ should be defined after twisting the corrugation, we can see that in the limit, $w \to 0$, $\mathbf{e}_{\mathrm{MN}}$ is parallel to $\mathbf{e}_{\mathrm{E'B}}$ (= $\mathbf{e}_{\mathrm{EB}}$). The twisting angle γ about axis $\mathbf{e}_{\mathrm{MN}}$ satisfies

$$\frac{\mathbf{e}_{\mathrm{D'F'}}}{\|\mathbf{e}_{\mathrm{D'F'}}\|}=\cos\gamma\frac{\mathbf{e}_{\mathrm{DF}}}{\|\mathbf{e}_{\mathrm{DF}}\|}+\sin\gamma\left(\frac{\mathbf{e}_{\mathrm{EB}}}{\|\mathbf{e}_{\mathrm{EB}}\|}\times\frac{\mathbf{e}_{\mathrm{DF}}}{\|\mathbf{e}_{\mathrm{DF}}\|}\right)+(1-\cos\gamma)\left(\frac{\mathbf{e}_{\mathrm{EB}}}{\|\mathbf{e}_{\mathrm{EB}}\|}\cdot\frac{\mathbf{e}_{\mathrm{DF}}}{\|\mathbf{e}_{\mathrm{DF}}\|}\right)\frac{\mathbf{e}_{\mathrm{EB}}}{\|\mathbf{e}_{\mathrm{EB}}\|}. \tag{20}$$

As shown in Fig. 2, we define the opening angles ψ and ψ' before and after twisting, respectively, such that

$$\cos\psi=\frac{\mathbf{e}_{\mathrm{DF}}}{\|\mathbf{e}_{\mathrm{DF}}\|}\cdot\frac{\mathbf{e}_{\mathrm{AC}}}{\|\mathbf{e}_{\mathrm{AC}}\|},\quad\text{and}\quad\cos\psi'=\frac{\mathbf{e}_{\mathrm{D'F'}}}{\|\mathbf{e}_{\mathrm{D'F'}}\|}\cdot\frac{\mathbf{e}_{\mathrm{AC}}}{\|\mathbf{e}_{\mathrm{AC}}\|}. \tag{21}$$

Taking into account that $\theta_1 = \theta_2$, we observe that

$$\cos\psi = 1 - \frac{4}{\cos\rho + 3}, \quad \text{and} \quad \lim_{w\to 0}\cos\psi' = \cos\theta_1 - \frac{2(1+\cos\theta_1)}{\cos\rho + 3}. \tag{22}$$

Now we multiply both sides of Eq. (20) by $(\mathbf{e}_{\mathrm{AC}}/\|\mathbf{e}_{\mathrm{AC}}\|)$. By doing so, we derive the following relationship:

$$\cos\psi' = \cos\gamma\cos\psi - (1-\cos\gamma)\left(\frac{2}{\cos\rho + 3}\right). \tag{23}$$

Substituting Eq. (22) into (23), we obtain the following identity in the asymptotic limit:

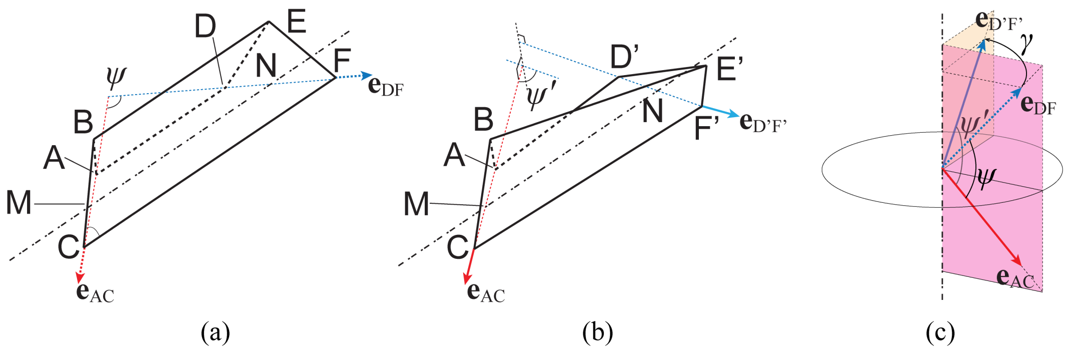

Supplementary Figure 2. Illustration of the ψ and ψ' angles. (a) A corrugation before twisting. (b) A corrugation after twisting. (c) Geometric relationship between ψ, ψ', and γ.

$$\cos\gamma = \cos\theta_1, \tag{24}$$

which directly leads to $\gamma = \theta_1 = \theta_2$. We use this result to simplify the expressions of ρ and ψ', and we get

$$\lim_{w\to 0}\cos\gamma = \cos^2(\rho/2), \quad \text{and} \quad \lim_{w\to 0}\cos\psi' = \cos\gamma - 1, \tag{25}$$

as presented in the main text. Notice that Eq. (25) are only necessary conditions to the compatibility constraints.

Supplementary Note 2: Establishment of the ODE

Setting a coordinate frame as shown in Fig. 2(a) of the main text, the piece of surface that conforms the geometry of the hypar folded shell in a quadrant can be parametrized as:

$$\mathbf{X}(r,t) = (1-t)[0,\ r,\ \zeta(r)] + t[r,\ 0,\ \xi(r)], \tag{26}$$

considering that

$$\boldsymbol{\xi}(r) = [r,\ 0,\ \xi(r)], \quad \text{and} \quad \boldsymbol{\zeta}(r) = [0,\ r,\ \zeta(r)]. \tag{27}$$

Based on observations of the physical model, we assume that the hypar origami surface is a graph, which has a unique projection onto the xy-plane. This parametrization of the global surface indicates that the projection of each loop of square crease onto the xy-plane remains a square but of a smaller size after folding, which is an assumption based on observation. In the geometric analysis, we find that $\triangle$ABC $\cong$ $\triangle$D'E'F' in the limit of $w \to 0$, which implies that $\boldsymbol{\zeta}(r)$ and $\boldsymbol{\xi}(r)$ have the same constituents at the outer rims, as demonstrated in Fig. 1(e). Thus we may assume that $\zeta(r) = -\xi(r)$, which leads to a simplified parametrization of the surface in the first quadrant as:

$$\mathbf{X}(r,t) = \left[tr,\ (1-t)r,\ (2t-1)\xi(r)\right], \quad r \geq 0,\ 0 \leq t \leq 1. \tag{28}$$

Indeed, we have reduced the problem of finding the shape of the meta-surface to establishing an ODE for the one dimensional function $\xi(r)$. To establish the ODE, we will use the previous geometric relationships derived locally for each corrugation. Due to the coordinate system we choose, the initial condition is $\xi(0) = 0$. We may also assume that ξ is a monotonically increasing convex function or monotonically decreasing concave function based on inspection of the actual shape of a folded hypar origami.

From a global view, we can define four tangent vectors at points M and N on the surface as follows:

$$\mathbf{X}_r^{\mathrm{M}} = \frac{\partial \mathbf{X}}{\partial r}(r,0) = [0,\ 1,\ -\xi'(r)] \tag{29}$$

$$\mathbf{X}_t^{\mathrm{M}} = \frac{\partial \mathbf{X}}{\partial t}(r,0) = [r,\ -r,\ 2\xi(r)] \tag{30}$$

$$\mathbf{X}_r^{\mathrm{N}} = \frac{\partial \mathbf{X}}{\partial r}(r,1) = [1,\ 0,\ \xi'(r)] \tag{31}$$

$$\mathbf{X}_t^{\mathrm{N}} = \frac{\partial \mathbf{X}}{\partial t}(r,1) = [r,\ -r,\ 2\xi(r)] \tag{32}$$

We remark that $\mathbf{X}_t^{\mathrm{M}} = \mathbf{X}_t^{\mathrm{N}}$, and they both align with the direction of $\mathbf{e}_{\mathrm{MN}}$. Computing the two surface normal vectors at M and N, we obtain:

$$\mathbf{n}^{\mathrm{M}} = \frac{\mathbf{X}_r^{\mathrm{M}} \times \mathbf{X}_t^{\mathrm{M}}}{\|\mathbf{X}_r^{\mathrm{M}} \times \mathbf{X}_t^{\mathrm{M}}\|} = \frac{[2\xi(r) - r\xi'(r),\ -r\xi'(r),\ -r]}{\sqrt{r^2 + r^2\xi'(r)^2 + (2\xi(r) - r\xi'(r))^2}}, \tag{33}$$

$$\mathbf{n}^{\mathrm{N}} = \frac{\mathbf{X}_r^{\mathrm{N}} \times \mathbf{X}_t^{\mathrm{N}}}{\|\mathbf{X}_r^{\mathrm{N}} \times \mathbf{X}_t^{\mathrm{N}}\|} = \frac{[r\xi'(r),\ -2\xi(r) + r\xi'(r),\ -r]}{\sqrt{r^2 + r^2\xi'(r)^2 + (2\xi(r) - r\xi'(r))^2}}. \tag{34}$$

The normals on two bounding curves may not be consistent for the two adjacent pieces of surface because there is no guarantee of first-order continuity at the joints. Therefore, we cannot assume that the x component of $\mathbf{n}^{\mathrm{M}}$ and the y component of $\mathbf{n}^{\mathrm{N}}$ are zeros. The discrete vectors $\mathbf{e}_{\mathrm{AC}}$ and $\mathbf{e}_{\mathrm{D'F'}}$ are approximations of $\mathbf{X}_r^{\mathrm{M}}$ and $\mathbf{X}_r^{\mathrm{N}}$, respectively. In addition, the twisting angle γ from the local analysis relates to the change between surface normals $\mathbf{n}^{\mathrm{M}}$ and $\mathbf{n}^{\mathrm{N}}$. Because both normals are orthogonal to $\mathbf{e}_{\mathrm{MN}}$, we write

$$\cos\gamma = \mathbf{n}^{\mathrm{M}} \cdot \mathbf{n}^{\mathrm{N}}. \tag{35}$$

Furthermore, using Eq. (25) and (21), we obtain

$$\cos\gamma = \frac{\mathbf{e}_{\mathrm{AC}} \cdot \mathbf{e}_{\mathrm{D'F'}}}{\|\mathbf{e}_{\mathrm{AC}}\|\|\mathbf{e}_{\mathrm{D'F'}}\|} + 1 = \frac{\mathbf{X}_r^{\mathrm{M}} \cdot \mathbf{X}_r^{\mathrm{N}}}{\|\mathbf{X}_r^{\mathrm{M}}\|\|\mathbf{X}_r^{\mathrm{N}}\|} + 1. \tag{36}$$

Equating the above two expressions, we obtain a first order ordinary differential equation (ODE):

$$\frac{(2\xi(r) - r\xi'(r))\,(2\xi(r) - 3r\xi'(r) - 2r\xi'(r)^3)}{(\xi'(r)^2 + 1)\left(r^2\left(\xi'(r)^2 + 1\right) + (2\xi(r) - r\xi'(r))^2\right)} = 0. \tag{37}$$

The denominator is always positive because $\xi(r)$ and $\xi'(r)$ are both real and positive. Thus the ODE can be simplified to:

$$(2\xi(r) - r\xi'(r))\left(2r\xi'(r)^3 + 3r\xi'(r) - 2\xi(r)\right) = 0. \tag{38}$$

This is the main result of this section. The solution of the governing ODE above is discussed in Eqs.(8) to (10) of the main text.

Supplementary Note 3: Origami mechanics explained by geometry

In the isometric deformation model, the system stored energy is contributed by the folding creases and bending hinges (introduced by triangulation). Therefore, we first need to associate angles of the local geometry with parameters of the global geometry. By Eqs. (36), (25), and (24), we obtain:

$$\cos\rho = \frac{1 - 4k^2r^2}{1 + 4k^2r^2}, \tag{39}$$

$$\cos\theta = \frac{1}{1 + 4k^2r^2}. \tag{40}$$

Let's denote ϕ as the angle between two consecutive diagonal creases, as shown in Fig. 3(a).

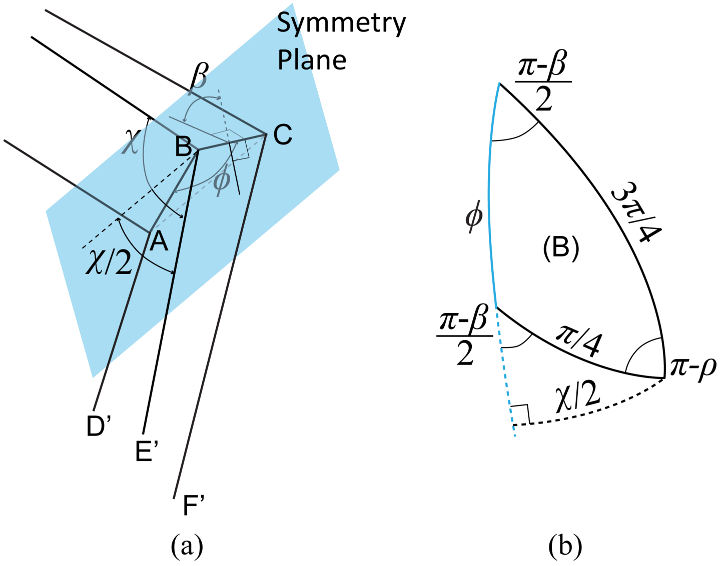

Supplementary Figure 3. Angles at a typical vertex. (a) Angles labeled in the direct geometry. (b) Angles labeled in the spherical representation at vertex B.

Applying spherical trigonometry at point B (see Fig. 3(b)), we obtain ϕ as a function of the folding angle ρ:

$$\cos\phi = \cos\frac{\pi}{4}\cos\frac{3\pi}{4} + \sin\frac{\pi}{4}\sin\frac{3\pi}{4}\cos(\pi-\rho) = -\frac{1}{1+4k^2r^2}. \tag{41}$$

Furthermore, *we denote the folding angles along the diagonal as* β, *and the angles between two square creases as* χ. Due to the symmetry of the folded shape, we compute $(\chi/2)$ and $(\beta/2)$ instead of χ and β (see Fig. 3):

$$\cos\frac{\chi}{2} = \mathbf{e}_{\mathrm{CA}}\cdot\mathbf{e}_{\mathrm{BE'}} \approx \mathbf{X}_t^{\mathrm{M}}\cdot\mathbf{X}_r^{\mathrm{M}} = \frac{4k^2r^2+1}{\sqrt{16k^4r^4+12k^2r^2+2}}, \tag{42}$$

$$\sin\frac{\beta}{2} = \frac{\sin(\chi/2)}{\sin(\pi/4)} = \frac{1}{\sqrt{2k^2r^2+1}}. \tag{43}$$

The degree-of-freedom (DOF) counting rule of origami patterns [3, 4] states that the generic DOF of a two-dimensional triangulated origami pattern (embedded in three-dimensional space) is given by the number of free boundary edges minus 3. The triangulated hypar kinematic model has four free boundary edges, therefore, once folded in a certain way (i.e., a kinematic branch is chosen), the triangulated hypar pattern has only one generic DOF. However, as we fold a hypar pattern, we cannot use Eqs. (11) and (13) from the main text to easily predict the trajectory of a certain point on the folded surface because both the quadratic coefficient k and the r-coordinate of a point (denoted as $r = R$) change

simultaneously, despite the fact that the folding kinematics is associated to a single DOF. This also makes it difficult to compare between different folded states. Let's denote the initial coordinate of a point on the diagonal creases as $(P, 0)$ on the flat configuration, and the current coordinate in the folded configuration as $(R, \xi(k, R))$. We would like to find a direct mapping between $(P, 0)$ and (R, ξ). Hence, we re-parametrize the curve ξ such that the current coordinate is written in terms of P as $(R(k, P), \tilde{\xi}(k, P))$. The derivation is explained below.

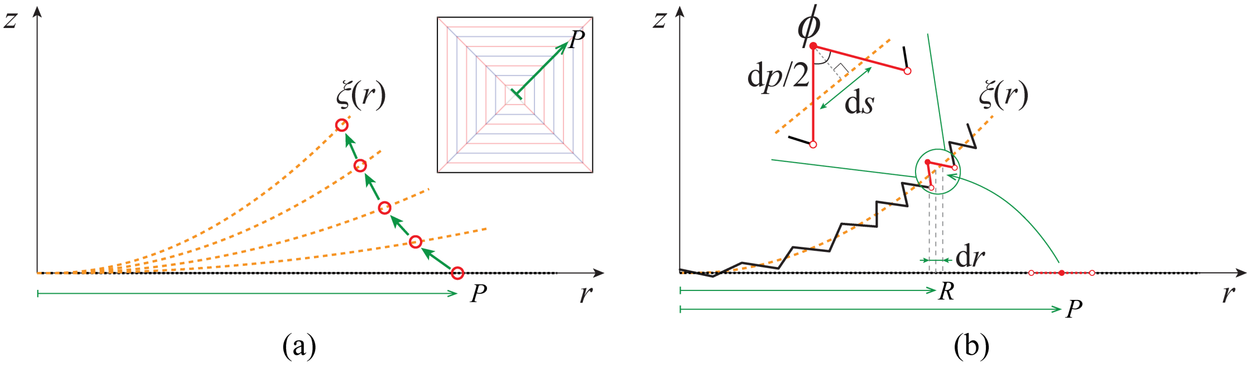

Supplementary Figure 4. The shape of ξ during folding. (a) The changing shapes of ξ at different folded states, and how a certain point on ξ changes its coordinate. (b) The local structure of ξ consists of a series of zig-zag folds of the diagonal creases.

Fig. 4(a) illustrates how the shape of ξ changes and how a certain point on ξ changes its coordinate. Recall that the local structure of ξ is constructed by zig-zags of the diagonal creases of the hypar pattern (see Fig. 4(b)). Assuming that each corrugation is infinitesimally thin, according to Fig. 4(b), we relate the differential length of diagonals (denoted as dp) with the differential arc length (denoted as ds) of ξ by:

$$\mathrm{d}p = \frac{1}{\sin(\phi/2)}\mathrm{d}s. \tag{44}$$

Furthermore, we relate dp with dr through ds [5]:

$$\mathrm{d}p = \frac{1}{\sin(\phi/2)}\mathrm{d}s = \frac{\sqrt{1+(\xi')^2}}{\sin(\phi/2)}\mathrm{d}r. \tag{45}$$

Integrating Eq. (45), we obtain the length of diagonal creases until the point $(R, \xi(R))$ on ξ as follows:

$$P = \int_0^R \frac{\sqrt{1+(\xi')^2}}{\sin(\phi/2)}\mathrm{d}r = \int_0^R \frac{4k^2r^2+1}{\sqrt{2k^2r^2+1}}\mathrm{d}r = R\sqrt{2k^2R^2+1}, \tag{46}$$

As we assume isometric deformation for the analytical model, the initial coordinate P is unchanged during folding for a point on the diagonal of a hypar pattern. Then we express R in terms of k and P as:

$$R(k,P) = \frac{1}{2}\sqrt{\frac{\sqrt{8k^2P^2+1}-1}{k^2}} \tag{47}$$

Since every value of R has a one-to-one correspondence with P, the shape of folded diagonal curve ξ can be redefined on the unfolded length of diagonals (denoted as p), according to a single changing geometric parameter k as:

$$\tilde{\xi}(k,p) = \xi(k, r(k,p)) = \frac{\sqrt{8p^2k^2+1}-1}{4k}. \tag{48}$$

The angles can also be expressed as functions of k and p, which are given by:

$$\rho(k,p) = \cos^{-1}\left(\frac{2}{\sqrt{8k^2p^2+1}} - 1\right), \tag{49}$$

$$\beta(k,p) = \pi - 2\sin^{-1}\left(\frac{\sqrt{2}}{\sqrt{1+\sqrt{8k^2p^2+1}}}\right), \tag{50}$$

$$\theta(k,p) = \cos^{-1}\left(\frac{1}{\sqrt{8k^2p^2+1}}\right). \tag{51}$$

With Eqs. (48) to (51), we can easily compare the positions of the points on the diagonal curve at different folded states.

Before we associate the system energy with the geometry of the folded hypar, we need to define a crease density function $\eta(p)$ such that:

$$n = \int_0^P \eta(p)\mathrm{d}p \tag{52}$$

where n is the number of square creases counted from the center until point P. Strictly, $\eta(p)$ is a summation of a series of shifted Dirac delta functions with values only at the locations of crease vertices along the diagonals (i.e. the line of p), which can be defined by:

$$\eta(p) = \sum_i^n \delta(p - p_i) \tag{53}$$

where δ is the Dirac delta function, and p_i's denote the locations of crease vertices. The strict definitions of n and η are shown in Fig. 5(a) and (b) by the solid lines, respectively. However,

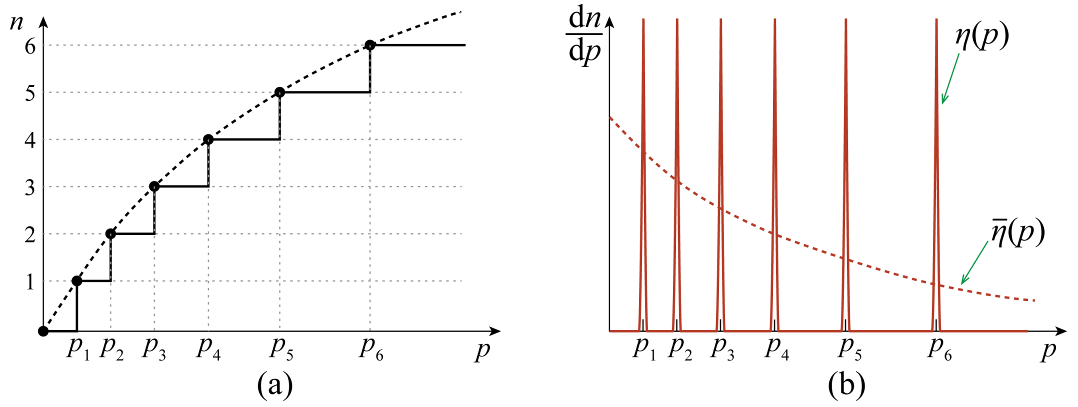

Supplementary Figure 5. The crease density function. (a) The crease counting function. The dots represents the actual data points. The solid line shows the strictly defined continuous function $n(p)$, while the dashed line shows the smoothed version of $n(p)$. (b) The crease density function $\eta(p)$ and homogenized crease density function $\bar{\eta}(p)$. The solid line refers to the strict definition, while the dashed line refers to the homogenized version.

such a discrete definition is usually difficult to construct for large patterns. Therefore, we propose a homogenized crease density function, which is defined as:

$$\bar{\eta}(p) = \frac{1}{\Delta p}\int_{p-\Delta p/2}^{p+\Delta p/2} \eta(q)\mathrm{d}q = \frac{\Delta n}{\Delta p}, \tag{54}$$

where Δp is taken as an arbitrary interval. The $\bar{\eta}(p)$ function can be interpreted as the rate of a smoothed function n changing over p, as shown in Fig. 5 by the dashed lines. We can first interpolate the data points of (n, p) by a smooth function, and then differentiate it to obtain $\bar{\eta}(p)$. For a standard hypar pattern with panel width d being a constant, $\bar{\eta}(p)$ is equal to the constant $(1/d)$. Because n cumulatively increases as p increases, then $\bar{\eta}(p)$ is always a positive function.

We associate the elastic energy of the system with its geometry. Suppose that the enforced folding has folded the pattern to a hyperbolic paraboloid with quadratic coefficient k_0, which defines the neutral angles of folding hinges. We define the stored energy of a folding hinge per unit length (i.e., the underlining constitutive model) as a convex function about the folding angle with the following properties:

$$\mathcal{H}_{\mathrm{F}}^{\rho}(\rho) \geq 0, \text{ with } \frac{\partial^2 \mathcal{H}_{\mathrm{F}}^{\rho}}{\partial \rho^2} > 0, \text{ and } \left.\frac{\partial \mathcal{H}_{\mathrm{F}}^{\rho}}{\partial \rho}\right|_{\rho=\rho_0} = 0, \tag{55}$$

where ρ_0 is the neutral (stress-free) angle of the folding hinge. Consequently, we know that

$$\frac{\partial \mathcal{H}_{\mathrm{F}}^{\rho}}{\partial \rho} < 0, \text{ when } \rho < \rho_0; \quad \text{and } \frac{\partial \mathcal{H}_{\mathrm{F}}^{\rho}}{\partial \rho} > 0, \text{ when } \rho > \rho_0. \tag{56}$$

A similar constitutive model can be defined for the stored energy of β (folding) angles, which is denoted as $\mathcal{H}_{\mathrm{F}}^{\beta}$. Thus,

$$\mathcal{H}_{\mathrm{F}}^{\beta} \geq 0, \ \frac{\partial^2 \mathcal{H}_{\mathrm{F}}^{\beta}}{\partial \beta^2} > 0, \ \left.\frac{\partial \mathcal{H}_{\mathrm{F}}^{\beta}}{\partial \beta}\right|_{\beta=\beta_0} = 0. \tag{57}$$

where we designate the neutral angle as β_0.

The constitutive model of a bending hinge is defined such that:

$$\mathcal{H}_{\mathrm{B}}^{\theta}(\theta) \geq 0, \text{ with } \frac{\partial^2 \mathcal{H}_{\mathrm{B}}^{\theta}}{\partial \theta^2} > 0, \text{ and } \left.\frac{\partial \mathcal{H}_{\mathrm{B}}^{\theta}}{\partial \theta}\right|_{\theta=\theta_0} = 0, \tag{58}$$

where θ_0 is the neutral (stress-free) angle of the bending hinge. Based on observation, the panels untwist when they are cut out from a folded hypar. Hence, we can assume that the neutral angles of bending hinges are at $\theta_0 = 0$, when all panels are flat (i.e. $k_0 = 0$). Thus, we obtain that:

$$\frac{\partial \mathcal{H}_{\mathrm{B}}^{\theta}}{\partial \theta} < 0, \text{ when } \theta < 0; \quad \text{and } \frac{\partial \mathcal{H}_{\mathrm{B}}^{\theta}}{\partial \theta} > 0, \text{ when } \theta > 0. \tag{59}$$

The simplest constitutive model for folding or bending hinges that satisfies our assumption is a quadric function. Please refer to Section Supplementary Note 4 of the Supplementary Information for examples.

Denote the total elastic energy of a hypar pattern as E_{T}, which is given by the summation of bending and folding energy:

$$E_{\mathrm{T}} = E_{\mathrm{F}} + E_{\mathrm{B}}, \tag{60}$$

for the triangulated model. Let P be the total length of diagonal creases from the center of the pattern, such that $(P, 0)$ is on the boundary of the pattern. The elastic energy stored in the folding hinges is computed as:

$$E_{\mathrm{F}} = 4 \int_0^P \bar{\eta} \left[(\sqrt{2}p) \mathcal{H}_{\mathrm{F}}^{\rho} + \mathcal{H}_{\mathrm{F}}^{\beta} \right] \mathrm{d}p \,, \tag{61}$$

where $(\sqrt{2}p)$ refers to the length of a square crease in the limit of $w \to 0$. We define the

neutral angles of ρ and β as the folded angles at state $k = k_0$, i.e.

$$\rho_0 = \cos^{-1}\left(\frac{2}{\sqrt{8k_0^2p^2+1}} - 1\right), \tag{62}$$

$$\beta_0 = \pi - 2\sin^{-1}\left(\frac{\sqrt{2}}{\sqrt{1+\sqrt{8k_0^2p^2+1}}}\right), \tag{63}$$

The elastic energy stored in the bending hinges is computed as:

$$E_{\mathrm{B}} = 4\int_0^P \bar{\eta}(\sqrt{2}p)\mathcal{H}_{\mathrm{B}}^{\theta}\mathrm{d}p\ , \tag{64}$$

where $(\sqrt{2}p)$ refers to the length of a bending crease in the limit of $w \to 0$.

Considering Eqs. (49) to (51), we note that the above expressions reveal the bistability of the folded hypar system. Since the term k always appears in its quadratic form (i.e. k^2), we conclude that a symmetry of system energy (with respect to k) exists for $k < 0$ and $k > 0$. In other words, for any folded state, there are different folded states of identical system energy, whose shape are defined by k of opposite sign but same magnitude. As a result, if we can show that there exist a local minimum of system energy of the hypar pattern for $k > 0$, the system is guaranteed to exhibit bistability.

Due to symmetry of system energy with respect to k, we only need to consider folded shapes with $k > 0$. We show below that there exist a local minimum of system stored energy for $0 < k < k_0$, which is true if we can find a k^* between 0 and k_0 such that:

$$\left.\frac{\partial E_{\mathrm{T}}}{\partial k}\right|_{k=k^*} = 0, \quad \text{and } \left.\frac{\partial^2 E_{\mathrm{T}}}{\partial k^2}\right|_{k=k^*} > 0. \tag{65}$$

Differentiating Eq. (61), we obtain:

$$\frac{\partial E_{\mathrm{F}}}{\partial k} = 4\int_0^P \bar{\eta}\left[(\sqrt{2}p)\frac{\partial \mathcal{H}_{\mathrm{F}}^{\rho}}{\partial \rho}\frac{\partial \rho}{\partial k} + \frac{\mathcal{H}_{\mathrm{F}}^{\beta}}{\partial \beta}\frac{\partial \beta}{\partial k}\right]\mathrm{d}p\ , \tag{66}$$

where,

$$\frac{\partial \rho}{\partial k} = \frac{8kp^2}{(8k^2p^2+1)\sqrt{\sqrt{8k^2p^2+1}-1}}, \tag{67}$$

$$\frac{\partial \beta}{\partial k} = \frac{\sqrt{2}}{k}\sqrt{\frac{\sqrt{8k^2p^2+1}-1}{8k^2p^2+1}}. \tag{68}$$

When $k = k_0$, we have $\rho = \rho_0$ and $\beta = \beta_0$, therefore, $(\partial\mathcal{H}_{\mathrm{F}}^{\rho}/\partial\rho) = 0$, and $(\partial\mathcal{H}_{\mathrm{F}}^{\beta}/\partial\beta) = 0$, which leads to $(\partial E_{\mathrm{F}}/\partial k) = 0$. When $k = 0$, we have $\rho < \rho_0$ and $\beta < \beta_0$, indicating that

$(\partial\mathcal{H}_{\mathrm{F}}^{\rho}/\partial\rho) < 0$ and $(\partial\mathcal{H}_{\mathrm{F}}^{\beta}/\partial\beta) < 0$. Because $\bar{\eta}$ is a positive function, we only need to examine the sign of $(\partial\rho/\partial k)$ and $(\partial\beta/\partial k)$ to determine the sign of $(\partial E_{\mathrm{F}}/\partial k)$. Since both Eqs. (67) and (68) are positive for $k > 0$, we know that $(\partial E_{\mathrm{F}}/\partial k) < 0$ for $0 < k < k_0$. When $k \to 0^+$, we obtain:

$$\lim_{k\to 0^+} \frac{\partial\rho}{\partial k} = 4p, \tag{69}$$

$$\lim_{k\to 0^+} \frac{\partial\beta}{\partial k} = 2\sqrt{2}p. \tag{70}$$

Hence, $(\partial E_{\mathrm{F}}/\partial k) < 0$ when $k \to 0^+$. On the other hand, differentiating Eq. (64) and taking $\theta_0 = 0$, we obtain:

$$\frac{\partial E_{\mathrm{B}}}{\partial k} = 4\int_0^P \bar{\eta}(\sqrt{2}p)\frac{\partial\mathcal{H}_{\mathrm{B}}^{\theta}}{\partial\theta}\frac{\partial\theta}{\partial k}\mathrm{d}p\ , \tag{71}$$

where,

$$\frac{\partial\theta}{\partial k} = \frac{2\sqrt{2}p}{8k^2p^2+1}. \tag{72}$$

We can see that $(\partial E_{\mathrm{B}}/\partial k) = 0$ when $k = 0$, and $(\partial E_{\mathrm{B}}/\partial k) > 0$ when $k > 0$. In conclusion, when $k \to 0^+$, $(\partial E_{\mathrm{F}}/\partial k) < (\partial E_{\mathrm{B}}/\partial k) = 0$; when $k = k_0$, $(\partial E_{\mathrm{B}}/\partial k) > (\partial E_{\mathrm{F}}/\partial k) = 0$.

Hence, we know that:

$$\left.\frac{\partial E_{\mathrm{T}}}{\partial k}\right|_{k\to 0^+} = \left.\frac{\partial E_{\mathrm{F}}}{\partial k}\right|_{k\to 0^+} < 0, \tag{73}$$

$$\left.\frac{\partial E_{\mathrm{T}}}{\partial k}\right|_{k=k_0} = \left.\frac{\partial E_{\mathrm{B}}}{\partial k}\right|_{k=k_0} > 0. \tag{74}$$

Because $(\partial E_{\mathrm{T}}/\partial k)$ is a continuous function, there must exist $0 < k^* < k_0$, such that:

$$\left.\frac{\partial E_{\mathrm{T}}}{\partial k}\right|_{k=k^*} = 0. \tag{75}$$

Furthermore, we let k^* be the smallest root of Eq. (75), i.e. the first intersection with 0. As $(\partial E_{\mathrm{B}}/\partial k) < 0$ when $k < k^*$, and $(\partial E_{\mathrm{B}}/\partial k) > 0$ when $k > k^*$, we know that the derivative of $(\partial E_{\mathrm{B}}/\partial k)$ must be positive at the point $k = k^*$. Therefore,

$$\left.\frac{\partial^2 E_{\mathrm{T}}}{\partial k^2}\right|_{k=k^*} > 0, \tag{76}$$

which indicates that at E_{T} has a local minimum at $k = k^*$.

The theory is based on the assumption that there exist convex stored energy functions for folding and bending hinges with different neutral states, i.e. the neutral angles of folding hinges correspond to a folded state, while the neutral angles of bending hinges correspond

to the flat (unfolded) state. We have proven that the bistability of the hypar pattern exists unconditionally, i.e. regardless of the specific constitutive models (i.e. $\mathcal{H}_{\mathrm{F}}^{\rho}$, $\mathcal{H}_{\mathrm{F}}^{\beta}$, and $\mathcal{H}_{\mathrm{B}}^{\theta}$), and crease offsets (i.e. η). In particular, the bistable behaviour of the hypar pattern is preserved, including the three cases shown in Fig. 1 and 4 of the main text, i.e. uniform, random, and functionally graded patterns.

Supplementary Note 4: Bar-and-hinge model for nonlinear numerical analysis

Here, we briefly describe the bar-and-hinge method that we used for the numerical modeling of hypar origami. The bar-and-hinge method is a tool for analyzing mechanical behavior of origami assemblages. Although it simplifies the kinematics of origami, the mechanics formulation is developed so that the simplified model can capture the essence of the origami behavior with non-rigid deformations [6–8]. The implementation in this paper is based on a nonlinear analysis formulation [8] coupled with the so-called N5B8 discretization scheme [7], which is explained below.

The basic idea of the N5B8 discretization is presented in Fig. 6. Each quadrilateral panel is divided into four triangles delimited by the diagonals, hence there are 5 nodes and 8 bars within each panel. Triangular panels are not further discretized. In a bar-and-hinge model, the one-dimensional stiffness of bars represents the in-plane stiffness of panels (e.g. stretching, shear). Out-of-plane stiffness (i.e. bending and folding) is simulated by rotational springs constraining each dihedral angle between two adjacent triangular frames. The N5B8 scheme allows the discrete system to capture doubly curved out-of-plane deformations and isotropic in-plane behavior of panels, yielding a much refined resolution [7] than the commonly adopted triangulation scheme [9–11] that simply divide each quadrilateral panel into two triangles.

We consider the discretized origami assemblage as an elastic system. The total strain energy (E_{T}) has contributions from the bars (E_{S}), bending hinges (E_{B}) and folding hinges (E_{F}). The total potential energy of the system is then:

$$E_{\mathrm{T}}(\mathbf{u}) = E_{\mathrm{S}}(\mathbf{u}) + E_{\mathrm{B}}(\mathbf{u}) + E_{\mathrm{F}}(\mathbf{u}) - \mathbf{f}^T\mathbf{u}, \tag{77}$$

where $\mathbf{f}$ is the externally applied load, and all the other energy terms are nonlinear functions of the nodal displacements $\mathbf{u}$. Equilibrium is obtained when E_{T} is locally stationary, and

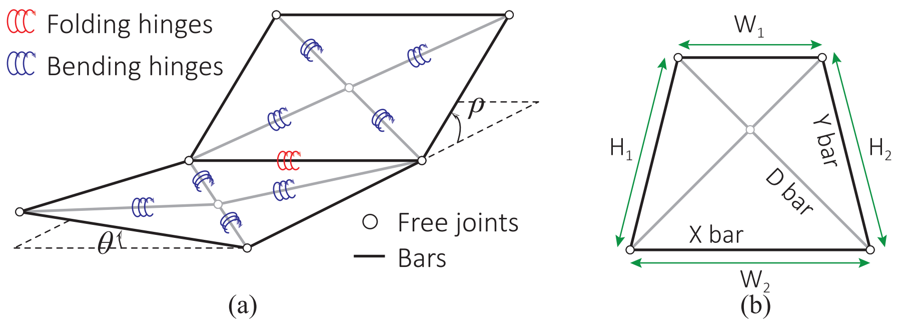

Supplementary Figure 6. Schematics of the N5B8 bar-and-hinge model for origami analysis. Interior edges (in gray) of panels are assigned with rotational springs representing bending deformations. Boundary edges (in black) shared with other panels are assigned with rotational springs representing folding deformations.

therefore the equilibrium equation and the finite element matrices can be derived as [7, 8]:

$$\mathbf{T}(\mathbf{u}) = \mathbf{T}_{\mathrm{S}}(\mathbf{u}) + \mathbf{T}_{\mathrm{B}}(\mathbf{u}) + \mathbf{T}_{\mathrm{F}}(\mathbf{u}) - \mathbf{f} = \mathbf{0}, \tag{78}$$

$$\mathbf{K}(\mathbf{u}) = \mathbf{K}_{\mathrm{S}}(\mathbf{u}) + \mathbf{K}_{\mathrm{B}}(\mathbf{u}) + \mathbf{K}_{\mathrm{F}}(\mathbf{u}), \tag{79}$$

where:

$$\mathbf{T}_{\mathrm{S}}(\mathbf{u}) = \frac{\partial E_{\mathrm{S}}(\mathbf{u})}{\partial \mathbf{u}},\ \mathbf{T}_{\mathrm{B}}(\mathbf{u}) = \frac{\partial E_{\mathrm{B}}(\mathbf{u})}{\partial \mathbf{u}},\ \mathbf{T}_{\mathrm{F}}(\mathbf{u}) = \frac{\partial E_{\mathrm{F}}(\mathbf{u})}{\partial \mathbf{u}}, \tag{80}$$

and

$$\mathbf{K}_{\mathrm{S}}(\mathbf{u}) = \frac{\partial^2 E_{\mathrm{S}}(\mathbf{u})}{\partial \mathbf{u}^2},\ \mathbf{K}_{\mathrm{B}}(\mathbf{u}) = \frac{\partial^2 E_{\mathrm{B}}(\mathbf{u})}{\partial \mathbf{u}^2},\ \mathbf{K}_{\mathrm{F}}(\mathbf{u}) = \frac{\partial^2 E_{\mathrm{F}}(\mathbf{u})}{\partial \mathbf{u}^2}. \tag{81}$$

The energy contribution for each deformation mode is the summation of elemental contributions, which is defined through elastic constitutive models.

For bar elements, we define the stored energy for a single bar as:

$$E_{\mathrm{S}} = AL\mathcal{W}(\varepsilon_{11}) \tag{82}$$

where A denotes member area, L denotes member length, and $\mathcal{W}$ is the energy density as a function of the one-dimensional Green-Lagrange strain ε_{11}. We adopt a one-dimensional Ogden model [12] for $\mathcal{W}$ such that

$$\mathcal{W}(\varepsilon_{11}) = \frac{Y}{\alpha_1 - \alpha_2}\left(\frac{\lambda_1(\varepsilon_{11})^{\alpha_1} - 1}{\alpha_1} + \frac{\lambda_1(\varepsilon_{11})^{\alpha_2} - 1}{\alpha_2}\right), \tag{83}$$

where Y is the modulus of elasticity, α_1 and α_2 are material constants taken as 5 and 1 [8], respectively. The principle stretch λ_1 is a function of ε_{11}, which is given by $\lambda_1 = \sqrt{2\varepsilon_{11} + 1}$

[13]. Accordingly, the 2nd Piola-Kirchhoff stress (the energy conjugate stress) is obtained as

$$S_{11} = \frac{\partial \mathcal{W}}{\partial \varepsilon_{11}} = \frac{\partial \mathcal{W}}{\partial \lambda_1}\frac{d\lambda_1}{d\varepsilon_{11}} = \frac{Y}{\alpha_1 - \alpha_2}\left(\lambda_1^{\alpha_1 - 2} + \lambda_1^{\alpha_2 - 2}\right). \tag{84}$$

For small strains, the constitutive model approximates linear elastic behavior, which occurs in our simulations as the strains of bar elements are very small ($< 1\%$). Denoting ν as the material's Poisson's ratio, to recover the in-plane Poisson's effect of the panel, we define the member areas as [7]:

$$\overline{A}_X = h\frac{\overline{H}^2 - \nu\overline{W}^2}{2\overline{H}(1-\nu^2)}, \quad \overline{A}_Y = h\frac{\overline{W}^2 - \nu\overline{H}^2}{2\overline{W}(1-\nu^2)}, \quad \overline{A}_D = h\frac{\nu(\overline{H}^2 + \overline{W}^2)^{3/2}}{2\overline{H}\overline{W}(1-\nu^2)}, \tag{85}$$

where $\overline{W} = (W_1 + W_2)/2$, $\overline{H} = (H_1 + H_2)/2$, and the subscripts X, Y, D label different bars, as indicated in Fig. 6(b).

The stored energy of each bending hinge is assumed to be a function of the hinge length (L) and bending angle (θ), which is given by

$$\mathcal{H}_{\mathrm{B}}^{\theta} = \frac{1}{2}LK_{\mathrm{B}}(\theta - \theta_0)^2, \tag{86}$$

where K_{B} denotes the bending stiffness constant. We assume K_{B} as [7, 14]:

$$K_{\mathrm{B}} = (1.0)\frac{Yh^3}{12(1-\nu^2)L_D}\left(\frac{L_D}{h}\right)^{1/3}, \tag{87}$$

where L_D is the total length of the diagonal on which the bending hinge lies. The scalar $(L_D/h)^{1/3}$ accounts for the scaling effect of ridge singularity [7, 15]. The resistance moment (or torque) of the hinge is then given by

$$\tau_{\mathrm{B}}^{\theta} = \frac{\partial \mathcal{H}_{\mathrm{B}}^{\theta}}{\partial \theta} = LK_{\mathrm{B}}(\theta - \theta_0). \tag{88}$$

We define the bending angle $\theta \in [-\pi, \pi)$, using absolute angles, such that $\theta = 0$ when the panel is flat. A bending hinge is stress-free when $\tau_b = 0$, that is, when $\theta = \theta_0$. In our implementation, the bending hinges are always assigned with $\theta_0 = 0$.

The stored energy of the folding hinges and their corresponding resistance moments are given by

$$\mathcal{H}_{\mathrm{F}}^{\rho} = \frac{1}{2}LK_{\mathrm{F}}(\rho - \rho_0)^2, \quad \tau_{\mathrm{F}}^{\rho} = LK_{\mathrm{F}}(\rho - \rho_0), \tag{89}$$

$$\mathcal{H}_{\mathrm{F}}^{\beta} = \frac{1}{2}LK_{\mathrm{F}}(\beta - \beta_0)^2, \quad \tau_{\mathrm{F}}^{\beta} = LK_{\mathrm{F}}(\beta - \beta_0), \tag{90}$$

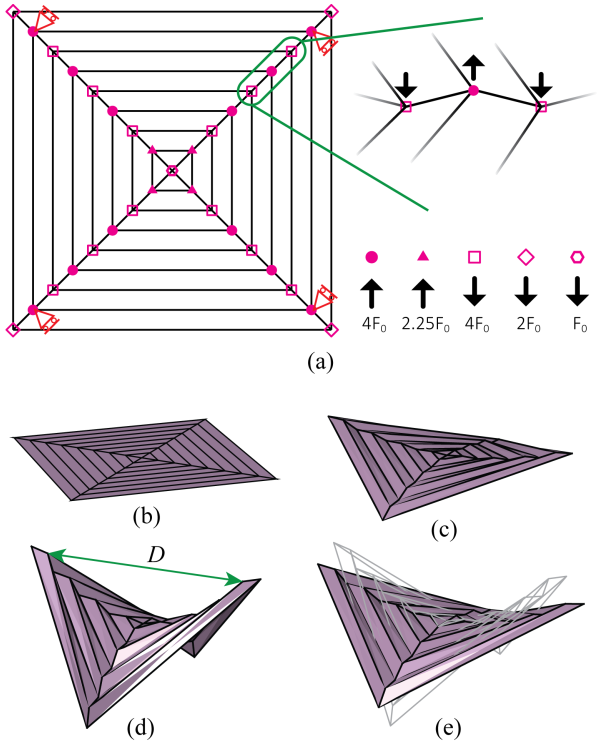

Supplementary Figure 7. Folding a numerical model of hypar origami. (a) The applied forces and kinematic constraints (red roller supports). Total upward and downward forces are balanced. The numbers are the relative magnitudes of forces normalized by a reference force F_0. (b)-(d) Frames along the folding process. The folding stops when distance D (as shown in green) becomes 80% of its original length when the sheet is flat (unfolded). (e) The new equilibrium shape after release of the folding forces and update of the neutral angles of folding hinges. The shape drawn in gray is the same as in (d), which shows the unbalanced configuration of the origami structure before the new equilibrium is found.

where K_F is the folding rotational stiffness, and ρ and β denote the folding angles. We assume $K_\mathrm{F} = \overline{K_\mathrm{B}}/2$, where $\overline{K_\mathrm{B}}$ denotes the average bending stiffness. *The stiffness reduction factor is taken as 2 based on the fact that we perforate the crease lines with equal distant slots that sum to half of the total crease length.*

To numerically fold a hypar pattern, we apply forces at the vertices to fold up the initially flat pattern, as shown in Fig. 7(a). A small symmetry-breaking perturbation is applied at the beginning of the loading to trigger one particular folding branch. In addition, because a flat pleated sheet has many singular deformation modes, it is easy to fold the pattern into undesired shapes. Thus, to improve the folding effectiveness, we reduce K_F further in this

step to distinguish the desired folding mode from other deformation modes. Please watch Supplementary Movie 3 for the folding simulation.

After the origami is folded to a hypar, we release the folding forces. To accommodate the inelastic deformation of folding creases within the nonlinear elastic framework, we update ρ_0 and β_0 after we release the folding forces so that the neutral angles of folding hinges are reset to the current folded state, as shown in Fig. 7(d). Meanwhile, the bending hinges still have their stress-free states at a flat configuration. As the other elements in the system remain elastic, after we release the folding forces, the system is unbalanced, and a new equilibrium must be found. This new equilibrium configuration then results from minimizing the combined energy (E) of folding (E_{F}), bending (E_{B}) and stretching (E_{S}) of the updated system. The configurational change before and after finding the new equilibrium is depicted in Fig. 7(e).

In Fig. 8, the color of the fold lines and the bend lines indicates the value of deformation angles, mapped onto the flat pattern of the hypar origami. Three different scenarios are considered: (1) the configurations at the end of enforced folding; (2) the equilibrium configurations of the updated models; (3) and the configurations after the snapping is finished, corresponding to frame 4 in Fig. 6(b) of the main text. We can clearly see that the dominant bending angles are forming the alternating asymmetric triangulation pattern in all cases.

Supplementary Note 5: Movie captions

Supplementary Movie 1 *Snapping of hypar origami (physical model)*

A Mylar based hypar origami display fast snapping between two stable and symmetric saddle configurations.

Supplementary Movie 2 *Snapping of hypar origami (numerical simulation)*

Numerical simulation using the bar-and-hinge reduced order model reveals the change of mechanical states during the bistable snapping of the hypar origami.

Supplementary Movie 3 *Folding of hypar origami (numerical simulation)*

Numerical model simulates the folding of the hypar origami from a flat sheet.

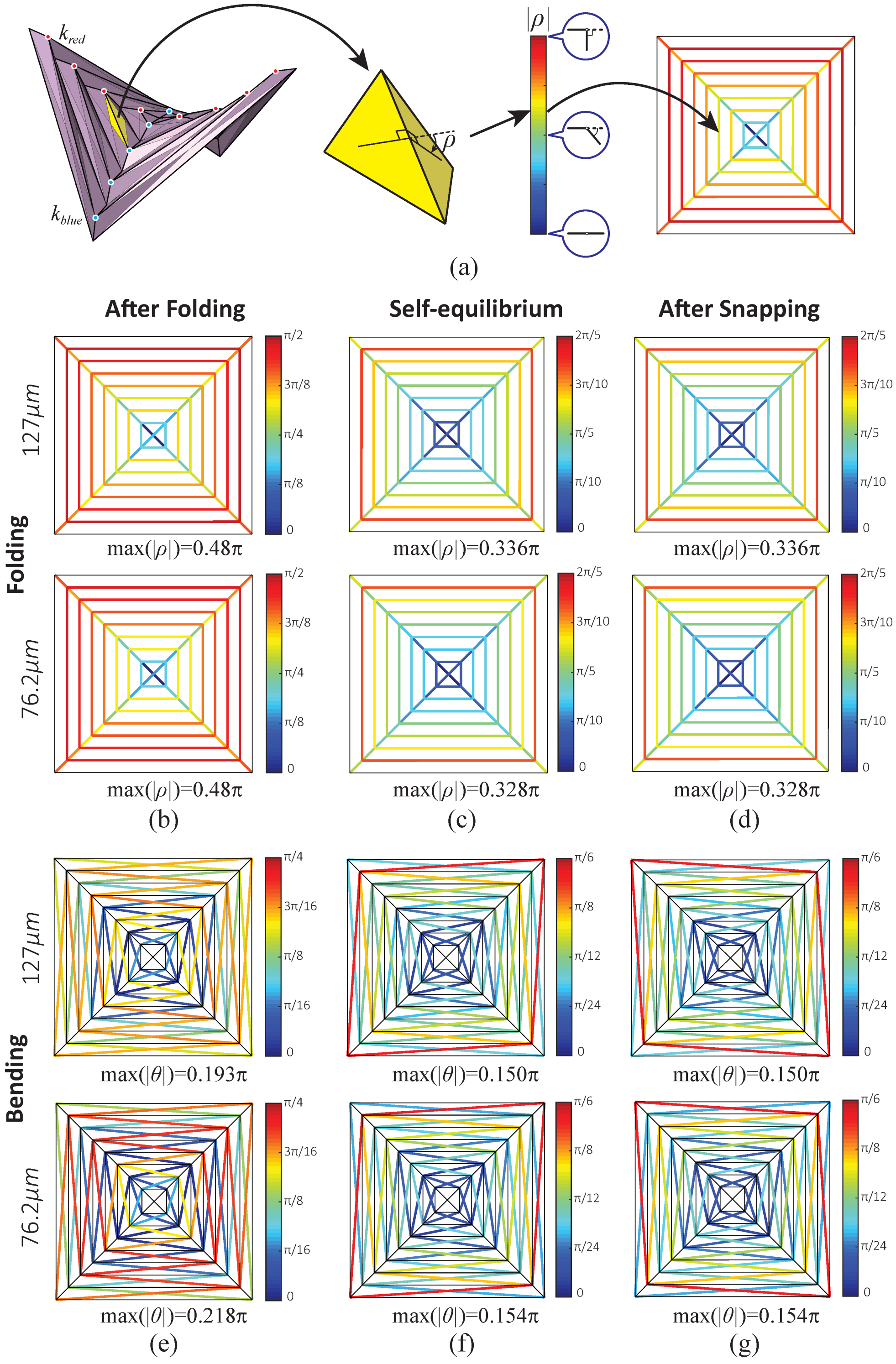

Supplementary Figure 8. Out-of-plane deformations (folding and bending) in terms of deformation angles (in absolute values). The average of k_{blue} and k_{red} interpolated by the blue and red dots are used as an estimation to the quadratic coefficient k in Eq. (13) of the main text.

Nomenclature

$\times$ Cross product

$\cdot$ Dot product

A_i Coordinate of point (vertex) A in the i-direction

d Panel width

L Corrugation length (=length of the middle folding ridge)

w Dimensionless panel width ($:= d/L$)

θ_1, θ_2 Bending angles

ρ, β Folding angles

ϕ Angles between two consecutive diagonal creases

$\mathbf{e}_{\mathrm{AB}}$ Vector pointing from point A to B

$\vec{n}$ Normals in the local geometry of a corrugation

$\mathbf{X}$ The surface approximated by a hypar origami

$\mathbf{X}_r, \mathbf{X}_t$ Tangents of the global surface

$\mathbf{n}$ Normals on the global surface

$\boldsymbol{\xi}, \boldsymbol{\zeta}$ Three-dimensional space curves

ξ, ζ One-dimensional curves

k, k_0 Quadratic coefficient of hyperbolic paraboloid geometry

E_{T} Total stored energy of a hypar origami structure

E_{S} Stretching energy

E_{B} Bending energy

E_{F} Folding energy

$\mathbf{T}$ Internal force vectors

$\mathbf{K}$ Stiffness matrices

$\mathbf{f}$ Applied forces on a bar-and-hinge model of an origami structure

$\mathbf{u}$ Nodal displacements of a bar-and-hinge model

$\mathcal{W}$ Strain energy density (of bar elements)

ε_{11} One dimensional component of the Green-Lagrange strain (of bar elements)

$\mathcal{H}$ Stored energy (of rotational spring elements)

Y Modulus of elasticity (initial)

ν Poisson's ratio

h Panel thickness

K_{B} Bending stiffness constant

K_{F} Folding stiffness constant

---

[1] Erik D. Demaine, Martin L. Demaine, Vi Hart, Gregory N. Price, and Tomohiro Tachi, "(non)existence of pleated folds: How paper folds between creases," Graphs and Combinatorics **27**, 377–397 (2011).

[2] John J. Craig, *Introduction to Robotics: Mechanics and Control*, 3rd ed. (Pearson, 2004).

[3] Tomohiro Tachi, "Freeform variations of origami," Journal for Geometry and Graphics **14**, 203–215 (2010).

[4] Bryan Gin-ge Chen and Christian D. Santangelo, "Branches of triangulated origami near the unfolded state," Physical Review X **8**, 011034 (2018).

[5] Manfredo P. Do Carmo, *Differential Geometry of Curves and Surfaces* (Prentice-Hall, 1976).

[6] Mark Schenk and Simon D Guest, "Origami folding: A structural engineering approach," in *Origami 5*, edited by Patsy Wang-Iverson, Robert J Lang, and Mark Yim (CRC Press, 2011) pp. 293–305.

[7] E. T. Filipov, K. Liu, T. Tachi, M. Schenk, and G. H. Paulino, "Bar and hinge models for scalable analysis of origami," International Journal of Solids and Structures **124**, 26–45 (2017).

[8] K. Liu and G. H. Paulino, "Nonlinear mechanics of non-rigid origami: an efficient computational approach," Proceedings of the Royal Society A **473**, 20170348 (2017).

[9] Z. Y. Wei, Z. V. Guo, L. Dudte, H. Y. Liang, and L. Mahadevan, "Geometric mechanics of periodic pleated origami," Physical Review Letters **110**, 215501 (2013).

[10] Mark Schenk and Simon D. Guest, "Geometry of Miura-folded metamaterials," Proceedings of the National Academy of Sciences **110**, 3276–3281 (2013).

[11] J. L. Silverberg, Jun Hee Na, A. A. Evans, B. Liu, T. Hull, C. D. Santangelo, Robert J. Lang, R. C. Hayward, and I. Cohen, "Origami structures with a critical transition to bistability arising from hidden degrees of freedom," Nature Materials **14**, 389–393 (2015).

[12] R. W. Ogden, *Non-Linear Elastic Deformations* (Dover Publications, New York, 1997).

[13] Peter Wriggers, *Nonlinear Finite Element Methods* (Springer, 2008) p. 559.

[14] Evgueni T. Filipov, Tomohiro Tachi, and Glaucio H. Paulino, "Origami tubes assembled into stiff, yet reconfigurable structures and metamaterials," Proceedings of the National Academy of Sciences **112**, 12321–12326 (2015).

[15] Alexander Lobkovsky, "Boundary layer analysis of the ridge singularity in a thin plate," Physical Review E **53**, 3750–3759 (1996).
