# Supplementary material for: Invariant and smooth limit of discrete geometry folded from bistable origami leading to multistable metasurfaces
Source: Nat Commun. 2019 Sep 17;10:4238. doi: 10.1038/s41467-019-11935-x (PMC6748981; doi:10.1038/s41467-019-11935-x)
Supplement: Supplementary file 2 — Description of Additional Supplementary Files [file 41467_2019_11935_MOESM2_ESM.docx]

Description of Additional Supplementary Files

**Supplementary Movie 1:** Snapping of hypar origami (physical model)

A Mylar based hypar origami display fast snapping between two stable and symmetric saddle configurations.

**Supplementary Movie 2:** Snapping of hypar origami (numerical simulation)

Numerical simulation using the bar-and-hinge reduced order model reveals the change of mechanical states during the bistable snapping of the hypar origami.

**Supplementary Movie 3:** Folding of hypar origami (numerical simulation)

Numerical model simulates the folding of the hypar origami from a flat sheet.
